# Muscle function mediates the association between vitamin D deficiency and hip fracture risk: a retrospective study in older adults

**DOI:** 10.3389/fendo.2025.1658529

**Published:** 2025-11-05

**Authors:** Xiongyi Wang, Yutong Hu, Simin Yun, Keyu Zhu, Miao Zheng, Qi Wei, Sheng Pan, Youjia Xu

**Affiliations:** Department of Orthopaedics, Second Affiliated Hospital of Soochow University, Osteoporosis Research Institute of Soochow University, Suzhou, China

**Keywords:** vitamin D, hip fractures, bone mineral density, osteoporosis, sarcopenia

## Abstract

**Objectives:**

Hip fracture, a critical public health concern in aging populations, are associated with significant morbidity, mortality, and disability and have been referred to as “the last fracture of life.” While vitamin D influences bone mineral density (BMD) and fragility fracture risk, its impact on muscle function and its relationship with fracture risk remains underexplored. This study aimed to investigate whether vitamin D deficiency increases hip fracture risk primarily through its effects on muscle function.

**Methods:**

In this retrospective study, 138 patients aged ≥ 50 years with initial low-energy hip fracture treated between January 2024 and June 2024 were compared with 182 community residents aged ≥ 50 years recruited from the hospital physical examination center. Clinical baseline data such as age, sex, and body mass index (BMI), were recorded. Through regression analysis, independent factors influencing hip fracture risk were analyzed. The effects of vitamin D on BMD and muscle function were evaluated using femoral neck areal BMD (FN aBMD) and pectoralis muscle index (PMI), respectively.

**Results:**

Patients with hip fracture were significantly older and had lower BMI, vitamin D level, muscle function, and FN aBMD than controls (P < 0.05). Univariate analysis identified age, BMI, vitamin D, PMI, and FN aBMD as key factors influencing hip fracture risk. After adjusting for age, sex and BMI, vitamin D, PMI, and FN aBMD emerged as independent protective factors against hip fracture in patients. Vitamin D was also found to be an independent protective factor against sarcopenia. However, Vitamin D levels did not significantly affect osteoporosis after adjusting for sex, age, and BMI. FN aBMD and PMI mediated 33.3% and 50.0%, respectively, of the association between vitamin D and HF.

**Conclusions:**

Vitamin D deficiency is associated with an increased risk of hip fracture, primarily through its impact on muscle function rather than BMD. Although vitamin D supplementation is crucial in older adults, integrating muscle function assessments into fracture prevention strategies is essential.

## 1 Introduction

Fracture is a significant public health issue, particularly in nations with aging populations. Among these, hip fracture (HF) is associated with high rates of morbidity, mortality and disability, earning them the moniker “the last fracture of life” (1–3). Consequently, it is crucial to identify individuals at high risk of HF and implement preventive measures as soon as possible.

Deficiency and insufficiency of vitamin D are prevalent in the elderly population (4). With aging, the capacity of the human skin to synthesize vitamin D decreases, as does the number of vitamin D receptors in human tissues (5). Furthermore, vitamin D deficiency in the elderly is compounded by limited exposure to sunlight due to factors such as physical frailty, mobility issues, or chronic illness (6, 7).

Current research on the effect of vitamin D on hip fracture risk is limited. Vitamin D, which is essential for the intestinal absorption of calcium, magnesium, and phosphate, plays a vital role in bone metabolism. Existing study indicated that vitamin D positively impacts intestinal calcium absorption and bone mineralization, ultimately reducing the fracture risk by increasing BMD (8). However, other studies suggest isolating vitamin D’s effects from calcium is challenging. Recent meta-analysis revealed that combining calcium with vitamin D effectively reduces hip and nonvertebral fractures by increasing BMD, whereas vitamin D alone does not produce the same effect (9). Moreover, a mendelian randomization study indicated that simply raising vitamin D levels do not significantly improve BMD in the general population (10).

Beyond BMD, physical activity and balance are critical in preventing falls and fractures. Beyond its role in bone metabolism, vitamin D is critically important for skeletal muscle. Vitamin D receptors (VDRs) are expressed in muscle cells, with vitamin D stimulates the proliferation and differentiation of myocytes (11). Deficiency in vitamin D is associated with sarcopenia. Sarcopenia is a progressive and widespread skeletal muscle disease with core features including:loss of skeletal muscle mass, loss of skeletal muscle strength, and loss of physical function (12). which are risk factors for falls (13, 14). Studies have shown that older adults with low vitamin D levels have poorer muscle function and a higher prevalence of sarcopenia, a condition that significantly contributes to fracture risk (15). We hypothesized that vitamin D deficiency increases the risk of falls and susceptibility to fractures primarily by affecting muscle function, rather than bone density.

Although the role of vitamin D in bone mineral density and calcium absorption is well-documented, its direct influence on muscle function as a primary mechanism for reducing hip fracture risk remains inadequately explored in clinical studies. Therefore, this study aims to investigate the strong association between vitamin D and HF and to determine whether vitamin D affects HF risk primarily through its effects on muscle or bone.

## 2 Methods

### 2.1 Study design and participants

**Figure 1:**
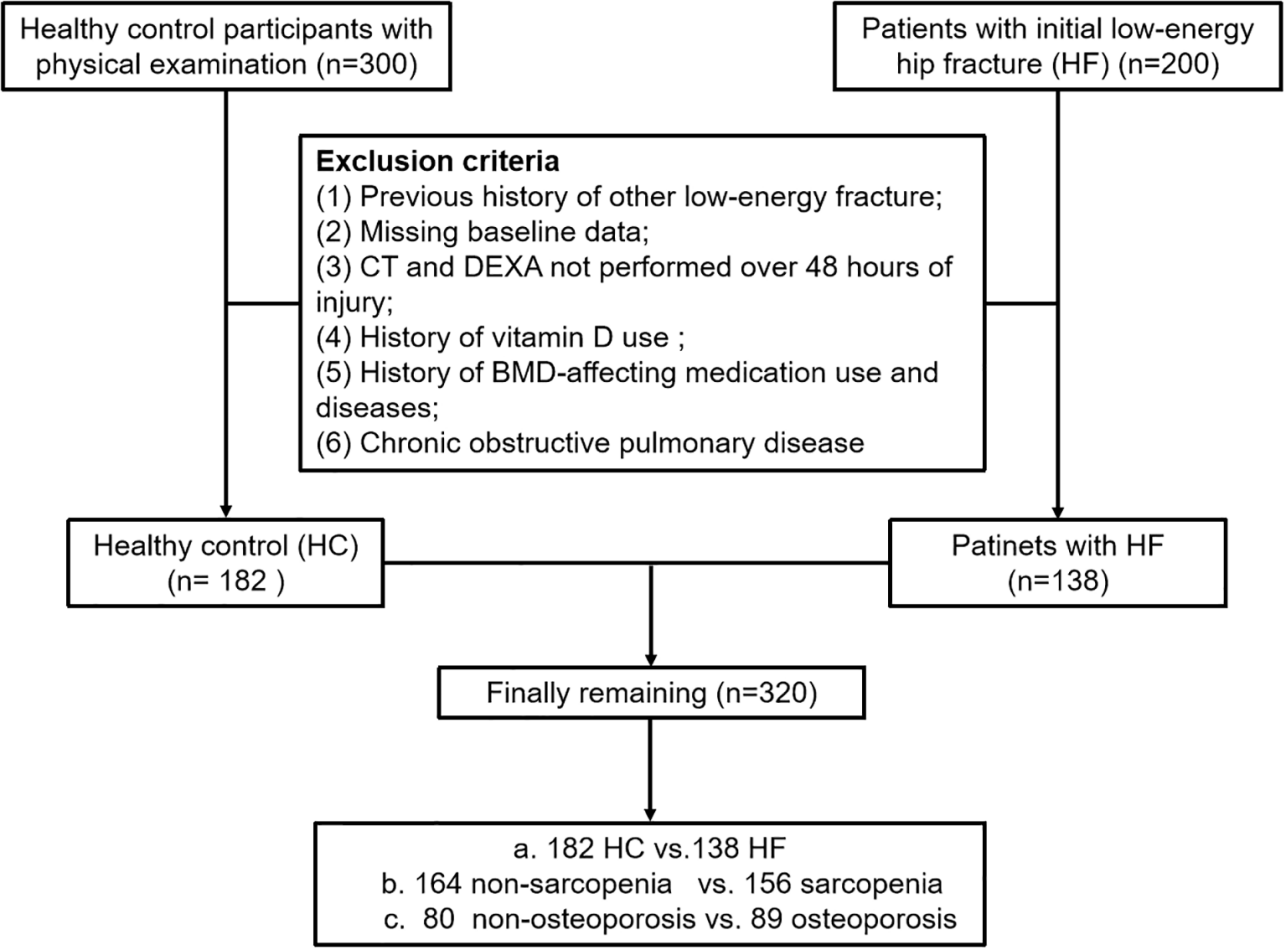
Study flowchart.

The study included 200 subjects aged ≥ 50 with initial low-energy HF from January 2024 to June 2024, designated as the HF group. Additionally, 300 community residents aged > 50 years were recruited from the Medical Examination Centre at the Second Affiliated Hospital of Soochow University as the healthy control (HC) group. To enhance comparability with the fracture cohort, the HC group was selected from non-fracture patients who underwent chest CT examinations at the same institution during the same period. Routine diagnostic procedures for HF patients included chest computed tomography (CT) and Dual-Energy X-ray Absorptiometry (DEXA). The same set of exclusion criteria was applied uniformly to both the fracture and non-fracture groups. Exclusion criteria were: 1. Previous history of other low-energy fracture (*e.g*,colles, vertebral, proximal humerus, etc.); 2. Missing baseline data; 3. Patients with fractures who do not undergo CT or DEXA scans within 48 hours of injury to minimize skeletal and muscular changes caused by post-fracture bed rest; 4. History of long-term use of calcium supplements and vitamin D supplements; 5. History of affecting-BMD medication use (*e.g.* bisphosphonates, parathyroid hormone and glucocorticoids, etc.) and diseases (*e.g.* rheumatoid arthritis and hyperthyroidism, etc.); 6. Chronic obstructive pulmonary disease. Lastly, A total of 320 participants were included in this study after applying the exclusion criteria (**Figure 1**). Relevant baseline data such as age, sex, and body mass index (BMI) were recorded.

This study has been approved by the Ethics Committee of the Second Affiliated Hospital of Soochow University. All participants provided written informed consent for participation. This study was conducted in accordance with the Declaration of Helsinki of the World Medical Association. Clinical trial number: not applicable.

### 2.2 Bone mineral density measurement

BMD was measured in all participants using DEXA (Lunar Prodigy dual-energy X-ray bone densitometer; GE Healthcare, Chicago, Illinois, USA). Measurements included BMD of the lumbar spine (L1-L4) and the hip, expressed as mg/cm^2^. The T-score was calculated based on the standard deviation from the mean BMD of a young and healthy population. The T-score is essential for diagnosing osteoporosis in post-menopausal women and men older than 50. According to WHO recommendation, osteoporosis was defined as T ≤ -2.5 in one of hip and spine, normal BMD as T ≥ -1.0, and osteopenia as -2.5 < T < -1.0 (16). In our study, participants with T-score ≤ -2.5 in one of hip and spine were classified as osteoporosis, while remaining participants were classified as non-osteoporosis.

### 2.3 Muscle measurement

**Figure 2:**
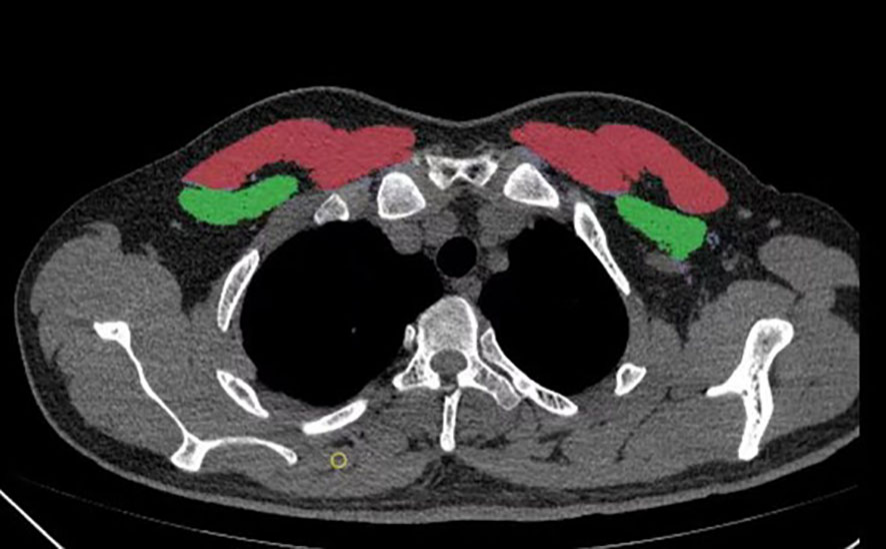
A method for determining pectoralis muscle area (PMA) by computed tomography (CT) with open-source software of 3D Slicer. a Horizontal plane of the T4 level; the pectoralis major is red, and the pectoralis minor is green.

A six-row spiral CT scanner (SOMATOM Emotion 6; Siemens, Munich, Germany) was used for chest CT scans. The scanning parameters included a tube voltage of 120 kV, automatic tube current adjustment, and a layer thickness of 0.625–2 mm. The CT images were analyzed with 3D Slicer, an open-source software application. The T4 level was defined as a middle slice of the fourth thoracic vertebra. The pectoralis major and minor muscles were manually segmented bilaterally. The bilateral pectoralis muscle area (PMA) was automatically calculated by summing the pixel attenuation within a threshold range of -29 to +150 HU for skeletal muscle (**Figure 2**). The pectoralis muscle index (PMI) was derived by dividing the PMA by the square of the height (cm^2^/m^2^). To assess inter-rater reliability, a second physician, blinded to the initial results, performed the measurements on a random sample of 100 subjects. Both measurements were performed by professionally trained physicians.

According to recent studies, sarcopenia is defined at the T4 level by a PMA of < 29.00 cm^2^ or PMI < 10.17 cm^2^/m^2^ in men, and a PMA of < 18.29 cm^2^ or PMI of < 7.31 cm^2^/m^2^ in women (17).

### 2.4 Vitamin D measurement

Fasting blood samples were collected at 8 am. Serum 25-hydroxy vitamin D (25(OH)D) levels were measured using an automated Roche electrochemiluminescence system (Roche Diagnostics GmbH, Mannheim, Germany). All procedures were performed according to the manufacturer’s standard protocols. Vitamin D deficiency was defined as 25(OH)D level of less than 20 ng/ml, and insufficiency as 20–30 ng/ml. Participants with 25(OH)D levels of more than 30 ng/ml were considered to have adequate vitamin D (18).

### 2.5 Statistical analysis

Statistical analyses were calculated by SPSS version 26.0. Participants were categorized into HF and HC groups. The normality of the data was tested with the Shapiro–Wilk test. Measurement data that conformed (or approximately conformed) to the normal distribution were expressed as mean ± standard deviation. The homogeneity of variance tests was simultaneously performed. Intergroup comparisons were performed using two independent sample t-tests when the variance of the variables of the two groups was homogeneous. Measurement data that did not conform to the normal distribution were presented as the median (inter quartile range) and compared using the Mann–Whitney U test. Furthermore, the counting data were expressed as frequency and percentage, and compared using chi-squared test. Univariate analysis was employed to identify potential risk factors for fractures, while multivariate analysis corrected for confounding variables. “RMediation” package in R 4.3.3. was utilized to perform Mediation analysis assessing the mediating effects of PMI and FN aBMD on the associations of Vitamin D with hip fracture (19). Statistical significance was set at P < 0.05.

## 3 Results

### 3.1 General characteristics of participants with HF and HC

**Figure 3:**
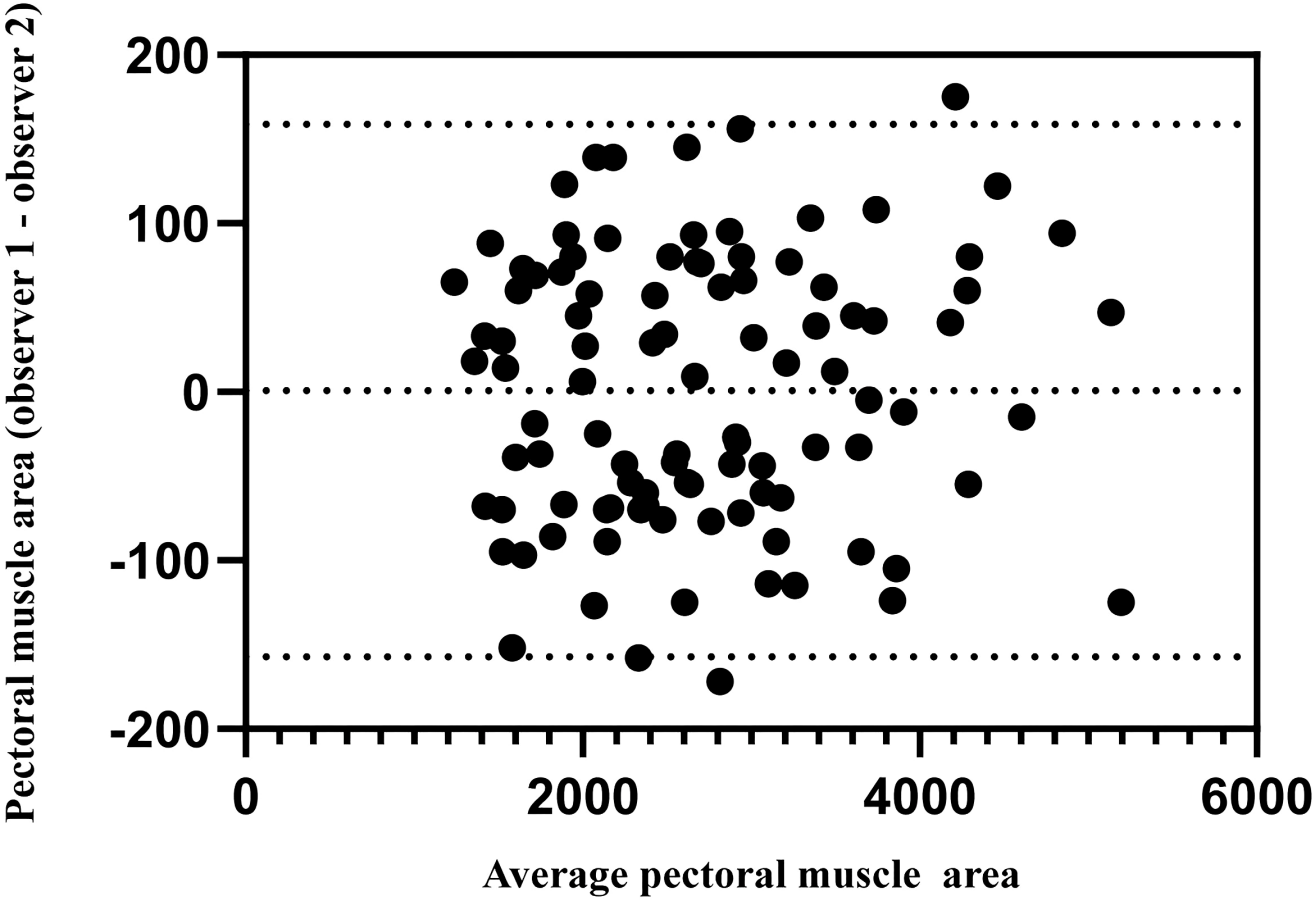
Bland–Altman plots for the interobserver variability.

The measurements of the two observers showed good agreement (ICC_PMA_ =0.992). **Figure 3** shows the interobserver variability of PMA measurements; the 95% limits of agreement in the Bland–Altman plot between the two observers ranged from -157.2 to 158.7 mm^2^ for PMA (**Figure 3**).

This study included 200 patients with initial low-energy hip fractures and 300 healthy control individuals. In the HF group, 62 participants were excluded: 18 with a previous history of other low-energy fracture, 11 due to missing baseline data, 10 for CT and DEXA more than 48 hours post-injury, 16 with a history of vitamin D or BMD-affecting medication use and diseases, 7 with chronic obstructive pulmonary disease. For the HC group, 118 participants were excluded. Ultimately, a total of 320 participants were included in the analysis, comprising 138 patients with HF and 182 HCs.

The general characteristics of the HF and HC groups are both shown in **Table 1**. The mean age of the HF group was higher than that of the HC (P < 0.001). BMI, vitamin D, PMA, PMI and BMD were significantly lower in the HF group (all P < 0.001). There were no significant differences in the sex distribution between the two groups (P = 0.45) (**Table 1**).

**Table 1:** Comparison of clinical baseline data in the HC and HF groups.

Characteristics	HC (n = 182)	HF (n = 138)	Mean difference	P
Sex, % (n)			n/a	0.45
Male	26.4 (48)	30.4 (42)		
Female	73.6 (134)	69.6 (96)		
Age (years)	66.01 ± 8.9	73.35 ± 9.0	-7.34	<0.001*
Weight (kg)	63.48 ± 10.2	57.67 ± 10.2	5.8	<0.001*
Height (cm)	157.0 (152.0, 161.0)	160 (155.0, 166.0)	-3	0.001*
BMI (kg/m^2^)	25.71 ± 3.6	22.51 ± 3.2	3.2	<0.001*
Vitamin D (ng/ml)	20.00 ± 7.5	15.78 ± 6.8	4.21	<0.001*
Muscle function				
PMA (cm^2^)	25.94 ± 8.0	17.5 ± 6.0	8.43	<0.001*
PMI (cm^2^/m^2^)	10.42 ± 2.8	6.79 ± 2.0	3.63	<0.001*
FN aBMD (mg/cm^2^)	835.83 ± 15.9	681.30 ± 113.4	154.53	<0.001*
Sarcopenia, % (n)	23.1 (42)	82.6 (114)	n/a	<0.001*
Osteoporosis grade, % (n)			n/a	<0.001*
normal	40.1 (73)	5.1 (7)		
osteopenia	45.1 (82)	50.0 (69)		
osteoporosis	14.8 (27)	44.9 (62)		

HC, healthy control; HF, hip fracture; BMI, body mass index; FN aBMD, femoral neck areal BMD; PMA, pectoralis muscle area; PMI, pectoralis muscle index.*Statistical significance at P< 0.05.

### 3.2 Partial correlation analyses

**Figure 4:**
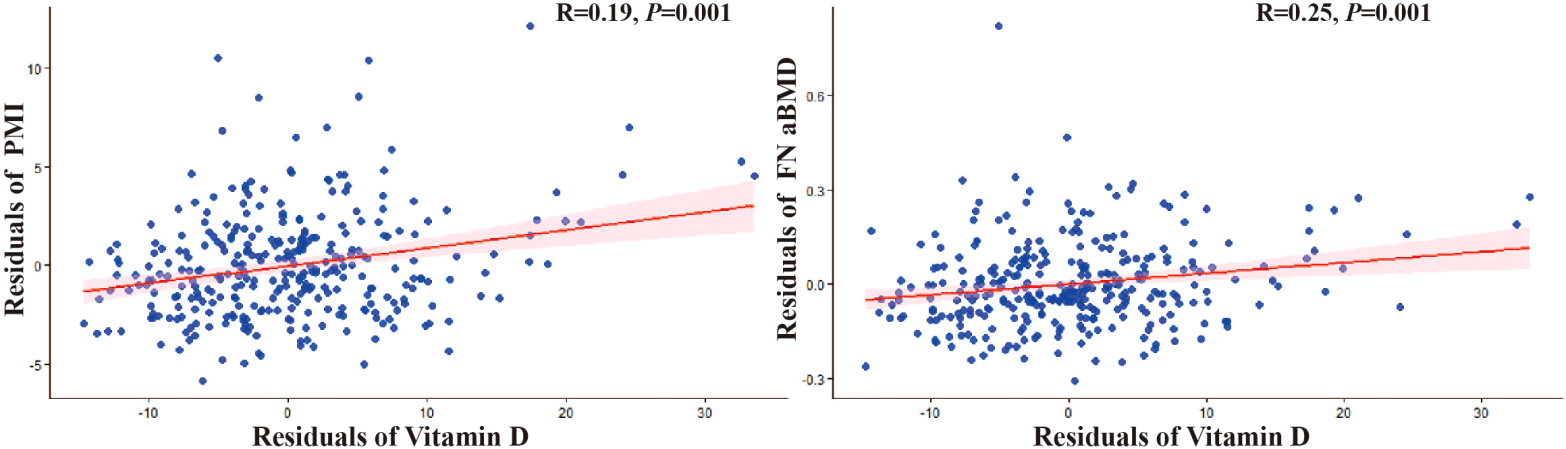
Partial correlation scatter plots of vitamin D with BMD and PMI.

To visually represent the key relationships independent of major confounders, we performed partial correlation analyses adjusting for age and BMI. **Figure 4** presents the partial regression scatterplots depicting the associations of serum vitamin D levels with PMI and BMD.

After adjusting for age and BMI, a significant positive partial correlation was observed between vitamin D and PMI (r = 0.25, p < 0.05). In contrast, the partial correlation between vitamin D and BMD, while also positive, was weaker (r = 0.19, p < 0.05).

### 3.3 Risk factors for HF

Univariate analysis identified that age, BMI, vitamin D level, PMI, and Femoral neck areal BMD (FN aBMD) were all significantly associated with hip fractures (all P < 0.001). Multivariate analysis, adjusted for sex, age, and BMI, revealed that vitamin D levels, PMI, and FN aBMD were independent protective factors against HF (all P < 0.001). For each 1ng/ml increase in vitamin D, the risk of hip fracture decreased to 0.91 times the original risk. Similarly, each 1cm^2^/m^2^ increase in PMI, the risk of hip fracture decreased to 0.45 times the original risk, while a 1mg/cm^2^ increase in FN aBMD decreased it to 0.99 times the original risk (**Table 2**).

**Table 2:** Univariate and multivariate logistic regression analysis of independent factors for HF.

Analysis and variable	OR	Adjusted OR	95% CI	P
Univariate analysis				
Age (years)	1.1		1.06-1.13	<0.001*
BMI (kg/m^2^)	0.75		0.69-0.81	<0.001*
Vitamin D (ng/ml)	0.92		0.88-0.95	<0.001*
PMI (cm^2^/m^2^)	0.50		0.43-0.59	<0.001*
FN aBMD (mg/cm^2^)	0.99		0.98-0.99	<0.001*
Multivariate analysis				
Vitamin D (ng/ml)		0.91	0.87-0.95	0.001*
PMI (cm^2^/m^2^)		0.45	0.36-0.56	<0.001*
FN aBMD (mg/cm^2^)		0.99	0.98-0.99	<0.001*

FN aBMD, femoral neck areal BMD; PMI, pectoralis muscle index.*Statistical significance at P< 0.05.

### 3.4 The relationship between vitamin D and muscle function

Participants were divided into sarcopenia and normal groups based on PMI and PMA values. **Table 3** compares the general characteristics of the two groups. Compared to the normal group, patients with sarcopenia were older and had lower BMI, vitamin D levels, and FN aBMD (all P < 0.001). The prevalence of osteoporosis was higher in the sarcopenia group, with no significant difference in sex distribution (**Table 3**).

**Table 3:** Comparison of clinical baseline data in the non- and sarcopenia groups.

Characteristics	Non-sarcopenia (n = 164)	Sarcopenia (n = 156)	Mean difference	P
Sex, % (n)			n/a	0.08
Male	44.4(40)	55.6(50)		
Female	55.6 (124)	44.4 (101)		
Age (years)	65.49 ± 9.0	73.04 ± 8.8	-7.55	<0.001*
Height (cm)	158.0 (153.0, 163.0)	158.0 (150.5, 165.0)	0	0.466
BMI (kg/m^2^)	25.84 ± 3.5	22.74 ± 3.4	3.10	<0.001*
Vitamin D (ng/ml)	19.14 ± 7.6	17.16 ± 7.3	1.98	0.018*
Muscle function				
PMA (cm^2^)	27.43 ± 7.4	16.91 ± 5.4	10.51	<0.001*
PMI (cm^2^/m^2^)	10.93 ± 2.6	6.68 ± 1.8	4.25	<0.001*
FN aBMD (mg/cm^2^)	830.97 ± 147.5	704.24 ± 137.4	126.73	<0.001*
Osteoporosis grade			n/a	<0.001*
normal	39.6(65)	9.6(15)		
osteopenia	50.0(82)	44.2(69)		
osteoporosis	10.4(17)	46.2(72)		

BMI, body mass index; FN aBMD, femoral neck areal BMD; PMA, pectoralis muscle area; PMI, pectoralis muscle index. *Statistical significance at P< 0.05.

After adjusting for sex, age, and BMI, vitamin D was identified as a protective factor against sarcopenia, reducing the risk by 0.96 times for each 1ng/ml increase in vitamin D levels (**Table 4**).

**Table 4:** Univariate and multivariate logistic regression analysis of independent factors for sarcopenia.

Analysis and variable	OR	Adjusted OR	95% CI	P
Univariate analysis				
Age (years)	1.10		1.07-1.13	<0.001*
BMI (kg/m^2^)	0.76		0.70-0.82	<0.001*
Vitamin D (ng/ml)	0.96		0.94-0.99	0.020l*
Multivariate analysis				
Vitamin D (ng/ml)		0.96	0.93-0.99	0.027*

BMI, body mass index. *Statistical significance at P< 0.05.

### 3.5 The relationship between vitamin D and BMD

Based on BMD, participants were classified into non-osteoporosis (n = 231) and osteoporosis (n = 89) groups. **Table 5** presents a comparison of their general characteristics. Osteoporosis patients were older and had lower proportions of men, BMI, PMA, and PMI (all P < 0.05). Sarcopenia prevalence was higher in the osteoporosis group. However, there was no significant difference in vitamin D levels between the osteoporosis and non-osteoporosis group (P = 0.07) (**Table 5**).

**Table 5:** Comparison of clinical baseline data in the non- and osteoporosis groups.

Characteristics	Non-osteoporosis (n = 231)	Osteoporosis (n = 89)	Mean difference	P value
Sex, % (n)			n/a	0.026*
Male	31.6 (73)	19.1 (17)		
Female	68.4 (158)	80.9 (72)		<0.001*
Age (years)	67.01 ± 9.0	74.78 ± 9.2	-7.76	<0.001*
Weight (kg)	63.66 ± 9.8	54.00 ± 9.3	9.67	<0.001*
Height (cm)	159.0 (155.0, 165.0)	155.0 (150.0, 160.0)	4.0	<0.001*
BMI (kg/m^2^)	25.09 ± 3.6	22.36 ± 3.5	2.73	<0.001*
Vitamin D (ng/ml)	18.65 ± 7.5	16.94 ± 7.5	1.71	0.07
Muscle function				
PMA (cm^2^)	24.52 ± 8.1	16.54 ± 6.0	7.98	<0.001*
PMI (cm^2^/m^2^)	9.63 ± 3.0	6.85 ± 2.4	2.78	<0.001*
Sarcopenia	36.4(84)	80.9(72)	n/a	<0.001*

BMI, body mass index; PMA, pectoralis muscle area; PMI, pectoralis muscle index. * Statistical significance at P< 0.05.

Multivariate analysis (**Table 6**) shows that vitamin D was not an independent predictor of osteoporosis after correcting for sex, age and BMI (P = 0.517).

**Table 6:** Univariate and multivariate logistic regression analysis of independent factors for osteoporosis.

Analysis and variable	OR	Adjusted OR	95% CI	P
Univariate analysis				
Age (years)	1.10		1.07-0.14	<0.001*
BMI (kg/cm^2^)	0.79		0.73-0.86	<0.001*
Vitamin D (ng/ml)	0.97		0.94-1.01	0.07
Multivariate analysis				
Vitamin D (ng/ml)		0.99	0.95-1.03	0.517

BMI, body mass index. *Statistical significance at P< 0.05.

### 3.6 Mediating role of PMI or FN aBMD

**Figure 5:**
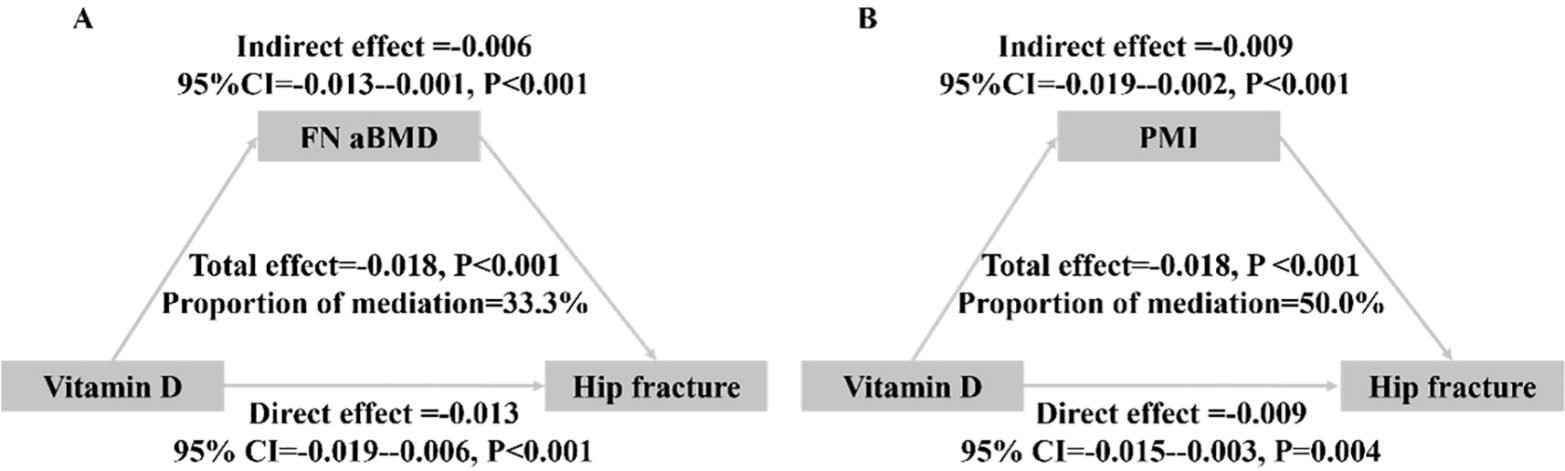
Analysis of the mediation by FN aBMD **(A)** and PMI **(B)** of the associations of Vitamin D with hip fracture.

As shown in **Figure 5**, FN aBMD mediated 33.3% of the association between Vitamin D and HF. Regarding the analysis of PMI, the proportion of mediation was 50.0%.

## 4 Discussion

This study investigated the effects of vitamin D on HF risk. We found that lower vitamin D level significantly increases the risk of HF. By exploring the relationship between vitamin D, BMD and muscle function, we demonstrated that reduced vitamin D levels increase HF risk primarily through effects on muscle function.

Previous studies have shown that fragility fractures predominantly occur in people over 50 years of age (20–23), therefore, our study focused on this age group. Muscle assessment is not routinely included in standard physical examinations. Based on previous studies that utilized a gender-based chest muscle index to assess the subjects’ muscle condition, we identified patients with sarcopenia through chest CT measurements of PMA and PMI, adhering to criteria validated in a large Asian population study (17).

Although previous studies have measured muscle area at the lumbar level, such as at the L4 vertebra, routine chest CT scans are widely used for the diagnosis and screening lung diseases while an abdominal CT is not (24). Over the past couple of years, the use of chest CT scans for routine physical examinations or pneumonia screening provided us with a vast amount of imaging data of chest. This makes the PMI assessment highly accessible and potentially valuable for clinical translation. Additionally, further studies (25) have shown that the PMI strongly correlates with BMD. The pectoralis muscle is a core muscle of the pectoral girdle and is crucial for maintaining upper trunk postural stability. Pectoralis muscle quality may serve as a valuable window into systemic muscle wasting. Wang et al. pointed out that muscle measurement in chest CT was associated with fracture. This correlation existed independently of bone density (26). Previous study (27) of our team also discovered that there exists a strong correlation between PMI from T4 level and hip fracture. To sum up, we finally selected pectoralis muscle area from T4 level in this study. Also, as the weakened function of primary respiratory muscles (such as the diaphragm) in patients with Chronic Obstructive Pulmonary Disease (COPD), accessory respiratory muscles like the pectoralis muscle may be recruited more frequently to sustain breathing. Although the overuse of the pectoralis major as an accessory respiratory muscle may initially cause muscle hypertrophy and a transient increase in PMI, chronic systemic muscle wasting, malnutrition, and decreased physical activity ultimately exacerbate muscle depletion, leading to a reduction in PMI over time (28). Therefore, to avoid the impact of COPD on PMI at different times, we excluded this group of participants.

Our findings indicate strong associations between vitamin D, BMD, muscle parameters, and HF risk. Patients with low vitamin D levels, osteoporosis, and sarcopenia are more susceptible to HF. Vitamin D remained an independent protective factor for HF after adjusting for sex, age, and BMI. Although the OR per 1-unit appears modest, the cumulative effect becomes substantial when considering changes over the physiological range of vitamin D levels. For instance, an increase in vitamin D from deficiency (e.g., 15 ng/mL) to sufficiency (e.g., 30 ng/mL) represents a 15-unit increase. The combined effect would be 0.91^15 ≈ 0.25. This suggests that improving vitamin D status from deficient to sufficient may be associated with an approximately 75% reduction in hip fracture risk, all else being equal. Prior research supports the crucial roles of the skeletal and muscular systems in fractures in older people (13, 29, 30). A randomized controlled trial showed that vitamin D supplementation improves muscle function in frail, elderly patients (31). Vitamin D’s role in bone health includes regulating calcium and phosphorus metabolism, with calcium deficiency contributing to osteoporosis. Consequently, vitamin D is a standard component in osteoporosis treatment guidelines (32). We hypothesize that vitamin D influences HF risk through its combined effects on the skeletal and muscular systems. However, there is still debate about which pathway has the greater impact.

Early clinical reports noted that reversible myopathy is frequently associated with severe vitamin D deficiency, suggesting a critical link (33). The biological basis for this is well-established: the role of vitamin D in muscle tissue is mediated by the abundant vitamin D receptors found in skeletal muscle cells (34). VDR expression in this tissue is critical for muscle cell activation and efficient vitamin D uptake. Active vitamin D stimulates metabolic processes in muscle tissue through nuclear and membrane receptors (35), promoting protein synthesis, shifting muscle fiber composition towards a higher proportion of fast-twitch, type II fibers, which are crucial for power and strength (36). Furthermore, vitamin D is essential for optimal calcium handling within muscle cells. It facilitates the influx of calcium ions across the cell membrane and influences the function of the sarcoplasmic reticulum, the main calcium store in muscle. This precise regulation of intracellular calcium is a prerequisite for the excitation-contraction coupling process, thereby directly enhancing the speed and force of muscle contractions (37). In short, through the above approaches, active vitamin D promotes muscle cell proliferation, inhibits apoptosis, and ultimately supports overall muscle homeostasis (38, 39). Therefore, vitamin D deficiency disrupts these vital processes, leading to a global decline in muscle function, including weakness, slowed contraction, and increased fatigue.

Sarcopenia, as a progressive, skeletal muscle disorder, affects mass, systemic strength, and balance (40). Decreased muscle function is strongly associated with adverse outcomes, including falls, fractures, disability, and mortality (41, 42). Consistent with these associations, our study found that sarcopenic patients had lower vitamin D levels than non-sarcopenic individuals. Vitamin D deficiency was an independent risk factor for sarcopenia after correcting for sex, age, and BMI. Thus, our findings suggest that vitamin D deficiency increases HF risk by impairing muscle function.

The causal role of vitamin D in bone metabolism is well established. The fundamental role of its active metabolites in intestinal calcium absorption and bone mineralization has long been known. The traditional paradigm posits that vitamin D enhances intestinal calcium absorption and bone mineralization, increases BMD, and reduces fracture risk. However, no clinical evidence supports the view that vitamin D supplementation reduces the risk of fractures mainly due to increased BMD (43). A cross-sectional study (44) showed that level of vitamin D and K in post-menopausal women was not associated with BMD, with only a negative correlation between vitamin D metabolites and parathyroid hormone (PTH). In a suburban Chinese population study, Gao et al. reported no correlation between level of vitamin D and lumbar spine or femoral neck BMD in post-menopausal women, with only a weak correlation observed for hip BMD (45). This may be explained by differences in laboratory batch measurements.

A Mendelian randomization study using Vitamin D Genome-Wide Association Study (GWAS) and whole-body BMD GWAS datasets by Sun et al. did not find a statistically significant association between genetically increased vitamin D levels and whole-body BMD (10). These results are compliance with recent randomized clinical trials and Mendelian randomization studies. (46, 47) Collectively, genetic analysis suggests that increased level of vitamin D do not significantly improve BMD in the general population. Consistent with these findings, our study observed no significant differences in vitamin D levels between osteoporosis and non-osteoporosis groups, further showing that it was not an independent risk factor for osteoporosis after adjusting for sex, age, and BMI. We propose that vitamin D’s influence on BMD plays only a minor role in HF risk, with the primary benefit related to its effects on muscle function.

There is no clear consensus on the specific effect of vitamin D on fracture risk. Several studies have reported Vitamin D supplementation did not result in a significantly lower risk of fractures among America adults who were not selected on the basis of vitamin D deficiency, low bone mass, or osteoporosis. The U.S. Preventive Services Task Force (USPSTF) does not recommend that community-living adults without osteoporosis or vitamin D deficiency take vitamin D supplements to prevent fractures (48). Besides, it has been suggested that the effects of supplemental vitamin D3 might be limited to those with low 25-hydroxyvitamin D levels. Higher prevalences of vitamin D deficiency have been reported among postmenopausal women and older persons with hip fractures (49, 50). Our real-world evidence supports the conclusion that vitamin D reduces HF risk primarily by improving muscle function rather than bone health.

However, this study has some limitations. First, as a retrospective study, we were unable to obtain direct functional measurements such as grip strength or gait speed, as these assessments are not part of the institution’s routine clinical process. Although PMI is feasible and reproducible, future prospective studies are needed to link imaging and functional data to validate the role of PMI as a surrogate marker of sarcopenia. Second, this study did not conduct age- and sex-stratified analyses, a limitation that should be addressed in future large-scale validation studies. Third, our study did not account for potential confounders such as the season of testing or habitual sunlight exposure. This limitation may have influenced the interpretation of the results. Future research should consider adjusting for these confounding factors. Finally, due to its retrospective nature and single-center design, which may limit the generalizability of the results. Future prospective, multi-center studies are needed to validate our findings.

## 5 Conclusion

In conclusion, vitamin D levels are associated with an increased HF risk. Our findings suggest that the association of vitamin D on BMD is minor, and that its contribution to reduced fracture risk may be primarily through its association with improved muscle function. Although vitamin D supplementation is critical for aging populations, assessing muscle function should be integrated into fracture prevention strategies. Future studies should explore the long-term impact of vitamin D supplementation on muscle function in younger populations and investigate whether targeted muscle-strengthening interventions combined with vitamin D therapy can further reduce fracture risk.

## Data Availability

The original contributions presented in the study are included in the article/**Supplementary Material**. Further inquiries can be directed to the corresponding authors.
